# Intra-tumor Genetic Heterogeneity and Mortality in Head and Neck Cancer: Analysis of Data from The Cancer Genome Atlas

**DOI:** 10.1371/journal.pmed.1001786

**Published:** 2015-02-10

**Authors:** Edmund A. Mroz, Aaron M. Tward, Rebecca J. Hammon, Yin Ren, James W. Rocco

**Affiliations:** 1 Center for Cancer Research and Division of Surgical Oncology, Massachusetts General Hospital, Boston, Massachusetts, United States of America; 2 Department of Otology and Laryngology, Harvard Medical School and Massachusetts Eye and Ear Infirmary, Boston, Massachusetts, United States of America; 3 The Broad Institute of MIT and Harvard, Cambridge, Massachusetts, United States of America; 4 Department of Otolaryngology–Head and Neck Surgery, Ohio State University, Columbus, Ohio, United States of America; Harvard Medical School, UNITED STATES

## Abstract

**Background:**

Although the involvement of intra-tumor genetic heterogeneity in tumor progression, treatment resistance, and metastasis is established, genetic heterogeneity is seldom examined in clinical trials or practice. Many studies of heterogeneity have had prespecified markers for tumor subpopulations, limiting their generalizability, or have involved massive efforts such as separate analysis of hundreds of individual cells, limiting their clinical use. We recently developed a general measure of intra-tumor genetic heterogeneity based on whole-exome sequencing (WES) of bulk tumor DNA, called mutant-allele tumor heterogeneity (MATH). Here, we examine data collected as part of a large, multi-institutional study to validate this measure and determine whether intra-tumor heterogeneity is itself related to mortality.

**Methods and Findings:**

Clinical and WES data were obtained from The Cancer Genome Atlas in October 2013 for 305 patients with head and neck squamous cell carcinoma (HNSCC), from 14 institutions. Initial pathologic diagnoses were between 1992 and 2011 (median, 2008). Median time to death for 131 deceased patients was 14 mo; median follow-up of living patients was 22 mo. Tumor MATH values were calculated from WES results. Despite the multiple head and neck tumor subsites and the variety of treatments, we found in this retrospective analysis a substantial relation of high MATH values to decreased overall survival (Cox proportional hazards analysis: hazard ratio for high/low heterogeneity, 2.2; 95% CI 1.4 to 3.3). This relation of intra-tumor heterogeneity to survival was not due to intra-tumor heterogeneity’s associations with other clinical or molecular characteristics, including age, human papillomavirus status, tumor grade and *TP53* mutation, and N classification. MATH improved prognostication over that provided by traditional clinical and molecular characteristics, maintained a significant relation to survival in multivariate analyses, and distinguished outcomes among patients having oral-cavity or laryngeal cancers even when standard disease staging was taken into account. Prospective studies, however, will be required before MATH can be used prognostically in clinical trials or practice. Such studies will need to examine homogeneously treated HNSCC at specific head and neck subsites, and determine the influence of cancer therapy on MATH values. Analysis of MATH and outcome in human-papillomavirus-positive oropharyngeal squamous cell carcinoma is particularly needed.

**Conclusions:**

To our knowledge this study is the first to combine data from hundreds of patients, treated at multiple institutions, to document a relation between intra-tumor heterogeneity and overall survival in any type of cancer. We suggest applying the simply calculated MATH metric of heterogeneity to prospective studies of HNSCC and other tumor types.

## Abstract 2

In this study, Rocco and colleagues examine data collected as part of a large, multi-institutional study, to validate a measure of tumor heterogeneity called MATH and determine whether intra-tumor heterogeneity is itself related to mortality.

## Editors’ Summary

**Background:**

Normally, the cells in human tissues and organs only reproduce (a process called cell division) when new cells are needed for growth or to repair damaged tissues. But sometimes a cell somewhere in the body acquires a genetic change (mutation) that disrupts the control of cell division and allows the cell to grow continuously. As the mutated cell grows and divides, it accumulates additional mutations that allow it to grow even faster and eventually from a lump, or tumor (cancer). Other mutations subsequently allow the tumor to spread around the body (metastasize) and destroy healthy tissues. Tumors can arise anywhere in the body—there are more than 200 different types of cancer—and about one in three people will develop some form of cancer during their lifetime. Many cancers can now be successfully treated, however, and people often survive for years after a diagnosis of cancer before, eventually, dying from another disease.

**Why Was This Study Done?:**

The gradual acquisition of mutations by tumor cells leads to the formation of subpopulations of cells, each carrying a different set of mutations. This “intra-tumor heterogeneity” can produce tumor subclones that grow particularly quickly, that metastasize aggressively, or that are resistant to cancer treatments. Consequently, researchers have hypothesized that high intra-tumor heterogeneity leads to worse clinical outcomes and have suggested that a simple measure of this heterogeneity would be a useful addition to the cancer staging system currently used by clinicians for predicting the likely outcome (prognosis) of patients with cancer. Here, the researchers investigate whether a measure of intra-tumor heterogeneity called “mutant-allele tumor heterogeneity” (MATH) is related to mortality (death) among patients with head and neck squamous cell carcinoma (HNSCC)—cancers that begin in the cells that line the moist surfaces inside the head and neck, such as cancers of the mouth and the larynx (voice box). MATH is based on whole-exome sequencing (WES) of tumor and matched normal DNA. WES uses powerful DNA-sequencing systems to determine the variations of all the coding regions (exons) of the known genes in the human genome (genetic blueprint).

**What Did the Researchers Do and Find?:**

The researchers obtained clinical and WES data for 305 patients who were treated in 14 institutions, primarily in the US, after diagnosis of HNSCC from The Cancer Genome Atlas, a catalog established by the US National Institutes of Health to map the key genomic changes in major types and subtypes of cancer. They calculated tumor MATH values for the patients from their WES results and retrospectively analyzed whether there was an association between the MATH values and patient survival. Despite the patients having tumors at various subsites and being given different treatments, every 10% increase in MATH value corresponded to an 8.8% increased risk (hazard) of death. Using a previously defined MATH-value cutoff to distinguish high- from low-heterogeneity tumors, compared to patients with low-heterogeneity tumors, patients with high-heterogeneity tumors were more than twice as likely to die (a hazard ratio of 2.2). Other statistical analyses indicated that MATH provided improved prognostic information compared to that provided by established clinical and molecular characteristics and human papillomavirus (HPV) status (HPV-positive HNSCC at some subsites has a better prognosis than HPV-negative HNSCC). In particular, MATH provided prognostic information beyond that provided by standard disease staging among patients with mouth or laryngeal cancers.

**What Do These Findings Mean?:**

By using data from more than 300 patients treated at multiple institutions, these findings validate the use of MATH as a measure of intra-tumor heterogeneity in HNSCC. Moreover, they provide one of the first large-scale demonstrations that intra-tumor heterogeneity is clinically important in the prognosis of any type of cancer. Before the MATH metric can be used in clinical trials or in clinical practice as a prognostic tool, its ability to predict outcomes needs to be tested in prospective studies that examine the relation between MATH and the outcomes of patients with identically treated HNSCC at specific head and neck subsites, that evaluate the use of MATH for prognostication in other tumor types, and that determine the influence of cancer treatments on MATH values. Nevertheless, these findings suggest that MATH should be considered as a biomarker for survival in HNSCC and other tumor types, and raise the possibility that clinicians could use MATH values to decide on the best treatment for individual patients and to choose patients for inclusion in clinical trials.

**Additional Information:**

Please access these websites via the online version of this summary at http://dx.doi.org/10.1371/journal.pmed.1001786.

- The US National Cancer Institute (NCI) provides information about cancer and how it develops and about head and neck cancer (in English and Spanish)
- Cancer Research UK, a not-for-profit organization, provides general information about cancer and how it develops, and detailed information about head and neck cancer; the Merseyside Regional Head and Neck Cancer Centre provides patient stories about HNSCC
- Wikipedia provides information about tumor heterogeneity, and about whole-exome sequencing (note that Wikipedia is a free online encyclopedia that anyone can edit; available in several languages)
- Information about The Cancer Genome Atlas is available
- A PLOS Blog entry by Jessica Wapner explains more about MATH

## Introduction

High intra-tumor heterogeneity has long been hypothesized to lead to worse clinical outcome [1–5]. Recent studies (reviewed in [6–11]) have documented the importance of intra-tumor heterogeneity in tumor development, metastasis, and treatment resistance.

One particularly important type of intra-tumor heterogeneity arises from differences among cancer cells that are inherited during cell division, which we refer to as genetic heterogeneity. Differences of a cancer cell’s genome from the germ line can result from unrepaired copy-number aberrations (CNAs) (amplification or loss of chromosomes, chromosome arms, or large genome segments) or smaller somatic mutations (single-nucleotide variants or short genomic insertions or deletions) that are passed on to a cell’s lineage during tumor development [12]. Even in a tumor originating from a single initiating clone, these processes can make the genome diverge among the tumor’s cancer cells, leading to cells with different CNA patterns [13] or to genetically distinct subclones [14]. This reservoir of genetically diverse cancer cells can promote metastasis or allow resistance to cytotoxic or molecularly targeted therapies [6–11].

Both CNAs and somatic mutations have been used to assess intra-tumor genetic heterogeneity and its relation to tumor development and patient outcomes. Gross CNAs seen via DNA staining in flow cytometry [15] or in fixed tissue [16] have long been associated with poor outcome. Intra-tumor differences in genome-segment amplification visualized by fluorescent in situ hybridization have been used to map the progression of breast cancers [17] and to suggest a mechanism for resistance to therapies targeted against individual receptor tyrosine kinases [18]. Analyses of CNA patterns or somatic mutations in different portions of the same tumor, even down to individual cells, have documented the importance of intra-tumor heterogeneity in tumor biology [19–22] and have supported the early hypothesis [1] that preexisting resistant subclones may be selected by therapy, leading to treatment failure [13].

The probable relation of intra-tumor genetic heterogeneity—whether from increased deviation from the germ line in cells with CNAs or from an increasing number of subclones—to poor outcome suggests that a measure of this heterogeneity would be a useful addition to cancer staging based on tumor node metastasis (TNM) [23] for prognosis. For patient care, improving prognostic accuracy would aid the development of clinical trials that stratify cancer patients according to likely outcomes under standard-of-care therapy; furthermore, patients whose tumors have low heterogeneity might be the best candidates for trials of targeted therapies or treatment de-intensification. Measurement of intra-tumor heterogeneity might thus eventually become an aid to clinical decision-making.

Incorporating information about intra-tumor genetic heterogeneity into clinical trial design and decision-making would best be done with a simple, quantitative, generally applicable measure. There have, however, been no large-scale studies on the relation of such a measure of intra-tumor heterogeneity to outcome for any type of cancer. Unfortunately, methods typically used in research on intra-tumor heterogeneity (reviewed in [24]) would be difficult to use widely in clinical research or practice. Pre-identified markers of tumor subpopulations [17,18] may not apply to tumor types other than those for which they were developed. Methods based on general CNA analysis are more widely applicable, but without multiple sampling of a tumor (as in [13]), they provide little information on intra-tumor heterogeneity caused by the presence of multiple subclones. Multiple sampling of individual tumors [13,22] or single-cell analysis [20,25] would be difficult to scale up for studies of hundreds of tumors or to assess in a timely fashion prior to beginning treatment. Methods that combine information on somatic mutations and copy-number changes to infer the subclonal composition of tumors [26–28] are still highly specialized, are computationally intensive, and typically require an underlying theoretical model or tumor-type-specific empiric examples of intra-tumor subclonal relations [29].

To overcome these difficulties, we recently developed a simple measure of intra-tumor genetic heterogeneity [30,31] based on whole-exome sequencing (WES) of tumor and matched normal DNA. With WES expected to play a significant role soon in clinical oncology [8,32], a measure of heterogeneity based on this technology could be widely used in practice. For each genomic locus having a tumor-specific mutation, WES provides the fraction of total sequenced DNA that shows the mutant allele, the mutant-allele fraction (MAF). We noted that the MAF value at any genomic locus would be influenced both by the presence of subclonal mutations and by CNAs. Typically, a locus mutated early in the clonal evolution of a tumor will be shared among later-arising subclones and have a high MAF in a bulk tumor specimen, while loci mutated later, restricted to one or a few subclones, have lower MAFs. Also, a mutation present on a DNA segment that has undergone allele-specific genomic amplification or loss should have a higher or a lower MAF, respectively, than a mutation at a locus that remains diploid. Because both subclonal mutations and CNAs would be expected to lead to differences in MAF values among genomic loci, we reasoned that the width of the distribution of MAF values among tumor-specific mutated loci within an individual tumor might capture intra-tumor genetic heterogeneity arising from both mechanisms. We thus proposed mutant-allele tumor heterogeneity (MATH), the width of this distribution (normalized by the median MAF value to correct for normal DNA in the tumor sample), as a simple quantitative measure of intra-tumor genetic heterogeneity [30].

In data from a prior study of patients with head and neck squamous cell carcinoma (HNSCC) [33], higher intra-tumor heterogeneity as measured by MATH from WES was related to worse outcome, particularly in patients who had received chemoradiotherapy [31]. Generalizability of this result, however, was limited, as all 74 patients came from a single institution. Furthermore, although human papillomavirus (HPV)–related HNSCC is of particular clinical interest because of its increasing incidence and improved clinical outcomes [34–36], only 11 cases in that dataset were HPV-positive. Consequently, although HPV-positive HNSCC tumors had significantly lower MATH values than HPV-negative tumors [30], we were limited in our ability to establish separate relations of MATH and HPV status to outcome because of the small sample size.

To examine whether this relation between intra-tumor heterogeneity and mortality could be generalized, we analyzed data on HNSCC from The Cancer Genome Atlas (TCGA) [37]. These open-access clinical and WES data provided an independent, large, multi-institutional validation dataset for testing the relation of MATH to clinical outcome. We examined the relation of MATH values to standard clinical variables, including HPV status, and to three molecular characteristics of HNSCC: mutation rate, *TP53* mutations [38], and oncogenic signature [12]. Using the same methods as in our previous work, we tested the hypothesis that intra-tumor heterogeneity, as measured by MATH, was related to mortality in patients with HNSCC after accounting for these potentially associated clinical and molecular characteristics.

## Methods

The de-identified, publicly available clinical data used in this study were those released by TCGA through October 8, 2013 [39]. The data tables downloaded on that date, for 360 patients, are provided as S1 Data. Initial pathologic diagnoses were between 1992 and 2011 (median, 2008). The TCGA head and neck consortium reported that the cases in the dataset were generally representative of a surgical case series of primary HNSCC, with T1 tumors underrepresented because of the tissue sample sizes needed for the multiple types of analyses performed on each tumor, and with most samples from the oral cavity or larynx [40,41]. Analysis of the data from the multiple contributing TCGA institutions and comparison against nationwide data support this characterization of the dataset (S1 Text).

For clinical data analysis, follow-up times and vital status reported in the main patient data table were updated from the follow-up tables. TNM classification was based on pathologic determination where available. Disease staging was as reported by TCGA. Radiation or chemotherapy delivered as primary therapy or adjuvant to surgery was distinguished from such therapy for recurrent disease or for palliation based on the “radiation_therapy,” “postoperative_rx_tx,” “targeted_molecular_therapy,” “regimen_indication,” and “regimen_indication_notes” fields in the TCGA patient, drug, radiation, and follow-up data tables. Absent a noted indication, radiation or chemotherapy was deemed primary/adjuvant if delivered within 180 d following initial pathologic diagnosis. As the University of Pittsburgh was the source of samples in our previous study [30,31], we verified that there was no overlap between the Pittsburgh cases we had already analyzed and the Pittsburgh TCGA cases.

Tumor-specific mutation data from WES were downloaded from the Broad Institute of MIT and Harvard [42], where WES had been performed [41]. Mutation data were available for 306 of the 360 patients with clinical data. To test and validate the results of our previous work [30,31] directly, we used identical methods for MATH analysis. The steps in determining the MATH value of an individual tumor from the WES data were (1) identifying genomic loci having tumor-specific somatic mutations, based on tumor–normal DNA comparisons; (2) tabulating the MAF (the fraction of DNA that shows the mutated allele at a locus) for mutated loci in that tumor; (3) determining the center and the width of the distribution of MAFs among those loci; and (4) taking the ratio of the width to the center of the distribution, expressed as a percentage.

Identification of tumor-specific mutations had already been performed at the Broad Institute of MIT and Harvard for TCGA, with the exome of tumor and matched normal DNA selected by Agilent SureSelect methods, followed by Illumina HiSeq sequencing. Mean sequence coverage was 95×, with 82% of bases in the targeted exome above 30× coverage [41]. The analysis pipeline was as in a previous study [33], with tumor-specific mutations for each case determined by the MuTect algorithm [43]. For each tumor, this algorithm uses the numbers of mutant and reference reads and the quality of the reads, in both tumor and patient-matched normal DNA, to estimate the likelihood that a particular locus has a tumor-specific rather than a germ-line mutation. Loci that pass the assigned threshold likelihood are deemed to have tumor-specific mutations. In total, 98.6% of mutant loci identified by MuTect in HNSCC and tested independently were validated [41].

The publicly available compilation of HNSCC mutant-allele data contained the numbers of WES reads showing the mutant allele and the number showing the reference allele at each tumor-specific mutated genomic locus for each tumor. We calculated the MAF for each locus as the ratio of mutant reads to total reads, and tabulated all MAF values for each tumor. For comparison with our earlier work [30,31], based on WES data with no reported MAF below 0.075 [33], we restricted analysis to genomic loci having MAFs at or above that value; no further restrictions were placed on the loci used for analysis. In one tumor, all mutations had MAF values below that cutoff, so 305 cases remained for this study.

For each tumor we then determined the median and the median absolute deviation (MAD) of its MAF values. The median is a robust measure of the center of the distribution of MAFs. The MAD is a robust measure of the width of the distribution that is much less sensitive to outliers than the standard deviation (SD), and is determined as follows: the absolute value of the difference of each MAF from the median MAF value is calculated, and the median of those absolute differences is taken. This median difference is then multiplied by a factor of 1.4826, so that the expected MAD of a normally distributed variable is equal to its SD.

Finally, the MATH value for each tumor was calculated as the percentage ratio of the MAD to the median of the distribution of MAFs among the tumor’s mutated genomic loci [30]: MATH = 100 × MAD/median. Simply using the width of the distribution as a measure of genomic heterogeneity would not take into account the overall lowering of MAF values by the “impurity” of normal DNA in the tumor sample. As previously described [30], dividing the MAD by the median provides a first-order correction for this “impurity,” as a more “impure” sample is expected to have a lower median MAF value.

**Fig 1:**
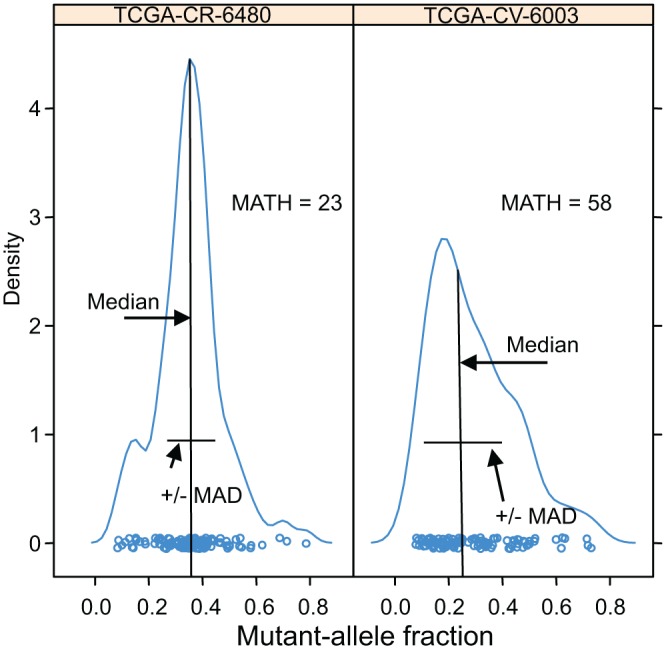
Examples of distributions of intra-tumor MAFs and their relation to MATH values. Density plots (smoothed histograms) of the distributions of MAFs for two HNSCCs. The horizontal-axis position of each circle represents the MAF for a tumor-specific mutated locus in the indicated tumor sample, for loci having MAFs no lower than 0.075: left, 117 loci; right, 106 loci. For each tumor, the median and the MAD of its MAFs are indicated; each tumor MATH value is the percentage ratio of its MAD to its median. The vertical density axis is scaled so that the area under the smoothed curve equals 1; a high peak density value indicates a sharp peak. MAFs of mutated loci in the high-heterogeneity tumor (right panel) show a lower median and higher MAD than those in the low-heterogeneity tumor (left panel), even though the total numbers of mutated loci, a measure of mutation rates within the tumors, are similar.

Examples of the distributions of intra-tumor MAF values and how these translate to the tumor’s MATH are shown for two cases in Fig. 1. In analyses that distinguished high- from low-heterogeneity tumors, we used the same cutoff value of 32 MATH units as in the previous study [31], without attempting to optimize the cutoff to the present data.

HPV status was based on the TCGA molecular classification, with tumor samples having more than 1,000 reads from RNA sequencing aligned to HPV sequences, or with evidence of genomically integrated HPV DNA, deemed HPV-positive [41]. A tumor was judged to have mutant *TP53* if it had any non-silent mutation in that gene. M-class and C-class oncogenic signatures were as reported by Ciriello et al. [12] for 267 of these tumors.

Relations of MATH values to other clinical and molecular characteristics were examined by linear models. Relations of MATH values and these characteristics to overall survival (time between initial pathologic diagnosis and death) were assessed by Cox proportional hazards analysis.

Receiver operating characteristic (ROC) curves for survival data were obtained by the nearest neighbor method of Heagerty et al. [44]. This method provides a smoothed estimate of the joint distribution of survival up to a chosen time and a continuous predictor variable. The ROC curve based on that distribution represents the tradeoff between specificity and sensitivity, in terms of survival predictions at the chosen time, as the value of the predictor variable (MATH in this case) is changed. Smoothing, by combining Kaplan-Meier survival analyses from cases that are neighbors in terms of the predictor variable, allows use of censored survival data, ensures a monotone relation between specificity and sensitivity, and makes ROC curves independent of monotone transformations of the predictor variable. We used a smoothing span of 0.1 (smoothing neighborhoods encompassing 10% of cases, except at the extremes of MATH values). Confidence intervals for the area under the curve (AUC) for ROC curves were estimated from bootstrap samples.

Calculations were performed in the R software environment [45], including its *survival*, *boot*, *rms*, and *survivalROC* packages. Significance analysis of hazard ratios (HRs) used the Wald test. Statistical significance was accepted at *p* < 0.05 in two-sided tests.

## Results

### Clinical Characteristics and Their Relations to Outcome

Among the 305 TCGA HNSCC patients with both clinical data and tumor MATH values, age ranged from 19 to 90 y, with a mean of 61.25, median of 61, SD of 12, and inter-quartile range of 16. Initial pathologic diagnoses were made between 1992 and 2011 (median, 2008). The median follow-up time for 174 patients still living at last record was 22.5 mo (overall range, 0 to 142 mo; inter-quartile range, 24.5 mo), and the median time to death for the other 131 patients was 14.3 mo (overall range, 0 to 211 mo; inter-quartile range, 16.4 mo). Thirty-six (12%) of the patients’ tumors were HPV-positive by TCGA molecular criteria.

Univariate relations of clinical characteristics to overall survival are shown in Table 1. Increased age, a history of smoking, higher T and N classifications, tumor grade, positive tumor margins, and presence of perineural invasion or of extracapsular spread from lymph nodes were all associated with diminished overall survival, and as expected [34,36,46,47], survival was much better for patients with HPV-positive versus HPV-negative tumors, with an overall survival HR of 0.34.

**Table 1:** Clinical and molecular characteristics, and their relations to overall survival.

Category	Characteristic	Number	Percent	Relation to Overall Survival		
				HR	95% CI	*p*-Value^#^
Clinical characteristics	Age			1.02/y	1.01–1.04	0.011
	<56 y	93	30.5			
	56 to 65 y	102	33.4			
	>65 y	110	36.1			
	Gender					
	Female	84	27.5	1		
	Male	221	72.5	0.8	0.55–1.15	0.22
	Race					
	White	264	86.6	1		
	American Indian/Alaska Native	1	0.3	No deaths		
	Asian	5	1.6	0.49	0.07–3.51	0.48
	Black/African American	28	9.2	1.6	0.94–2.70	0.08
	NA	7	2.3			
	Ethnicity					
	Hispanic/Latino	14	4.6	1		
	Not Hispanic/Latino	281	92.1	0.85	0.39–1.83	0.68
	NA	10	3.3			
	Alcohol use (drinks per day)					
	<1	24	7.9	1		
	1 to 3	53	17.4	1.65	0.73–3.74	0.23
	>3	43	14.1	1.65	0.69–3.98	0.26
	NA	185	60.7			
	Tobacco history					
	Never smoked/quit >15 y	109	35.7	0.48	0.32–0.71	<0.001
	Current smoker/quit ≤15 y	186	61.0	1		
	NA	10	3.3			
	Prior cancer diagnosis					
	No	288	94.4	1		
	Yes	17	5.6	0.86	0.38–1.97	0.73
	Neoadjuvant history					
	No	296	97.0	1		
	Yes	9	3.0	1.44	0.69–2.99	0.33
	Tumor site					
	Oral^†^	185	60.7	1.8	0.94–3.48	0.078
	Oropharynx^†^	39	12.8	1		
	Larynx	78	25.6	1.7	0.84–3.45	0.14
	Hypopharynx	3	1.0	1.88	0.24–14.7	0.55
	T classification*			1.22/class	1.02–1.46	0.031
	1	24	7.9	1		
	2	92	30.2	1.55	0.60–3.98	0.36
	3	81	26.6	2.67	1.05–6.77	0.039
	4	108	35.4	2.21	0.88–5.55	0.091
	Tumor histologic grade					
	G1	25	8.2	1		
	G2	187	61.3	2.17	1.08–4.37	0.03
	G3	79	25.9	1.48	0.71–3.12	0.30
	G4	4	1.3	No deaths		
	GX/NA	10	3.3			
	Tumor margins					
	Negative	213	69.8	1		
	Close	20	6.6	1.41	0.71–2.81	0.33
	Positive	27	8.9	1.72	1.02–2.90	0.042
	NA	45	14.8			
	Perineural invasion					
	Absent	101	33.1	1		
	Present	106	34.8	1.78	1.14–2.76	0.011
	NA	98	32.1			
	Lymphovascular invasion					
	Absent	133	43.6	1		
	Present	65	21.3	1.4	0.89–2.20	0.14
	NA	107	35.1			
	N classification*			1.20/class	1.00–1.44	0.045
	0	130	42.6	1		
	1	40	13.1	0.88	0.50–1.58	0.68
	2	126	41.3	1.33	0.91–1.94	0.14
	3	8	2.6	2.67	1.14–6.24	0.024
	NA	1	0.3			
	TNM stage*			1.20/stage	0.99–1.46	0.067
	I	17	5.6	1		
	II	53	17.4	2.12	0.63–7.06	0.22
	III	48	15.7	2.4	0.72–8.05	0.15
	IV	187	61.3	2.71	0.86–8.59	0.09
	Extracapsular spread					
	None	136	44.6	1		
	Microscopic	37	12.1	2.36	1.42–3.92	0.001
	Gross	18	5.9	1.75	0.91–3.38	0.096
	NA	114	37.4			
	HPV status					
	Negative	242	79.3	0.34	0.16–0.70	0.003
	Positive	36	11.8	1		
	NA	27	8.9			
Tumor molecular characteristics	Number of exome mutations			1.44/10-fold	0.90–2.30	0.13
	<90	101	33.1			
	90 to 150	107	35.1			
	>150	97	31.8			
	*TP53* status					
	Wild-type	92	30.2	1		
	Mutant	213	69.8	2.61	1.67–4.07	<0.001
	Oncogenic signature					
	C-class	193	63.3	1		
	M-class	96	31.5	0.62	0.42–0.92	0.018
	NA	16	5.2			

^#^ *p*-Values for Wald test in univariate Cox proportional hazards analysis, omitting NA cases.

^†^Oral also includes oral tongue, lip, alveolar ridge, buccal mucosa, floor of mouth, and hard palate; oropharynx also includes base of tongue and tonsil.

*Results shown both for relation to outcome as numeric variable, and for individual categories.

NA, not available.

HPV-positive HNSCC is now considered to be a different type of disease from HPV-negative HNSCC [36]. As shown in S1 Table, HPV status was significantly related to many clinical characteristics. Adjusting for HPV status did not affect the statistical significance of most relations between clinical characteristics and overall survival (S2 Table). Exceptions were T classification (no longer significant) and TNM stage (reached significance when analyzed as a numeric variable adjusted for HPV status; S2 Table). These relations between clinical variables and outcome are expected in HNSCC [46], and support the clinical relevance of these TCGA data.

We examined whether there were differences among the institutions that contributed tissue samples and patient data to TCGA. As demonstrated in S1 Text, most of the apparent differences in survival among institutions could be attributed to different mixes of patient characteristics among institutions, particularly in terms of HPV status.

### MATH Values and Their Relation to Clinical Characteristics

**Fig 2:**
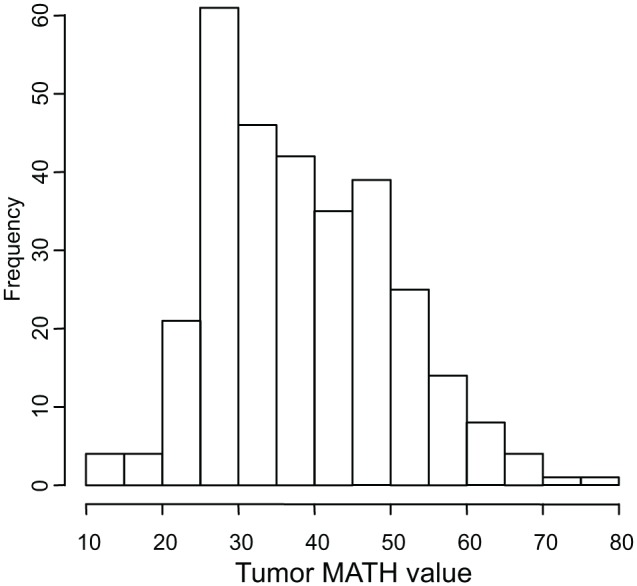
Histogram of MATH values among 305 HNSCCs. Horizontal axis, MATH values; vertical axis, number of tumors having MATH values within the indicated ranges.

Tumor MATH values ranged from 12.0 to 77.3, with a median of 37.0, a mean of 38.4, and first and third quartiles of 29.0 and 46.4, respectively. The distribution of MATH values among tumors is shown in Fig. 2.

**Fig 3:**
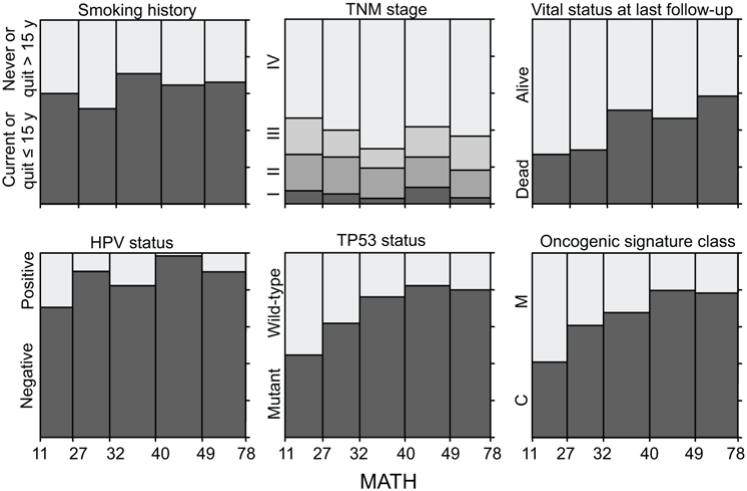
Relations of selected clinical and molecular characteristics to MATH values. Each panel represents the relation of a clinical or molecular characteristic to MATH values. Within each panel, the horizontal axis represents the range of tumor MATH values, divided into five groups with approximately equal numbers of tumors. The relative heights of the shaded portions within each vertical bar indicate the proportions of cases having the indicated characteristics.

Relations between MATH values and clinical characteristics were assessed by univariate linear models in which MATH value was the outcome variable and each clinical characteristic was taken individually as a predictor variable. MATH value was significantly related to tumor site, tumor grade, presence of lymphovascular invasion (LVI), and a history of prior cancer diagnosis or neoadjuvant therapy (Table 2). It was also highly related to tumor HPV status, validating our prior report [30]. The relations of selected patient clinical characteristics and tumor molecular characteristics to MATH values are displayed in Fig. 3.

**Table 2:** Relations of clinical and molecular characteristics to MATH values.

Category	Characteristic	Number	Percent	Relation to MATH Value	
				MATH ± SD^#^	*p*-Value^†^
Clinical characteristics	Age*			−0.075/y ± 0.057	0.19
	<56 y	93	30.5	39.4 ± 12.5	
	56 to 65 y	102	33.4	37.8 ± 12.3	
	>65 y	110	36.1	38.0 ± 10.9	
	Gender				0.27
	Female	84	27.5	37.1 ± 9.9	
	Male	221	72.5	38.8 ± 12.5	
	Race				0.13
	White	264	86.6	37.8 ± 11.7	
	American Indian/Alaska Native	1	0.3	49.4	
	Asian	5	1.6	35.3 ± 12.0	
	Black/African American	28	9.2	42.8 ± 12.4	
	NA	7	2.3	40.0 ± 13.8	
	Ethnicity				0.32
	Hispanic/Latino	14	4.6	41.3 ± 11.9	
	Not Hispanic/Latino	281	92.1	38.0 ± 12.0	
	NA	10	3.3	43.1 ± 8.7	
	Alcohol use (drinks per day)				0.75
	<1	24	7.9	37.8 ± 12.4	
	1 to 3	53	17.4	37.2 ± 11.3	
	>3	43	14.1	39.2 ± 13.8	
	NA	185	60.7	38.5 ± 11.6	
	Tobacco history				0.25
	Never smoked/quit >15 y	109	35.7	37.2 ± 11.6	
	Current smoker/quit ≤15 y	186	61.0	38.9 ± 12.2	
	NA	10	3.3	40.1 ± 8.3	
	Prior cancer diagnosis				0.009
	No	288	94.4	37.9 ± 11.7	
	Yes	17	5.6	45.6 ± 13.6	
	Neoadjuvant history				0.002
	No	296	97.0	38.0 ± 11.5	
	Yes	9	3.0	50.6 ± 16.6	
	Tumor site				0.040
	Oral^‡^	185	60.7	37.9 ± 10.8	
	Oropharynx^‡^	39	12.8	34.9 ± 14.3	
	Larynx	78	25.6	41.2 ± 12.7	
	Hypopharynx	3	1.0	34.8 ± 11.8	
	T classification*			+1.33/class ± 0.69	0.055
	1	24	7.9	35.9 ± 11.8	
	2	92	30.2	36.6 ± 11.1	
	3	81	26.6	39.6 ± 12.0	
	4	108	35.4	39.3 ± 12.3	
	Tumor histologic grade				0.001
	G1	25	8.2	33.4 ± 10.9	
	G2	187	61.3	39.7 ± 11.8	
	G3	79	25.9	37.9 ± 10.9	
	G4	4	1.3	20.8 ± 11.1	
	GX/NA	10	3.3	36.7 ± 16.1	
	Tumor margins				0.90
	Negative	213	69.8	38.7 ± 10.6	
	Close	20	6.6	38.3 ± 11.9	
	Positive	27	8.9	39.7 ± 12.9	
	NA	45	14.8	35.7 ± 12.3	
	Perineural invasion				0.74
	Absent	101	33.1	39.2 ± 12.3	
	Present	106	34.8	38.7 ± 10.6	
	NA	98	32.1	37.1 ± 12.8	
	LVI				0.005
	Absent	133	43.6	37.5 ± 11.1	
	Present	65	21.3	42.4 ± 11.8	
	NA	107	35.1	36.9 ± 12.4	
	N classification*			+0.62/class ± 0.70	0.38
	0	130	42.6	37.5 ± 12.4	
	1	40	13.1	39.2 ± 11.8	
	2	126	41.3	38.9 ± 11.4	
	3	8	2.6	38.1 ± 13.8	
	NA	1	0.3	50.0	
	TNM stage*			+0.40/stage ± 0.72	0.58
	I	17	5.6	38.5 ± 11.8	
	II	53	17.4	37.2 ± 11.8	
	III	48	15.7	38.5 ± 12.9	
	IV	187	61.3	38.6 ± 11.7	
	Extracapsular spread				0.11
	None	136	44.6	37.3 ± 11.4	
	Microscopic	37	12.1	41.3 ± 10.2	
	Gross	18	5.9	40.9 ± 15.3	
	NA	114	37.4	38.2 ± 12.3	
	HPV status				0.004
	Negative	242	79.3	39.8 ± 11.2	
	Positive	36	11.8	33.9 ± 13.5	
	NA	27	8.9	31.2 ± 12.2	
Tumor molecular characteristics	Number of exome mutations*			+3.75/10-fold ± 1.83	0.041
	<90	101	33.1	35.6 ± 12.7	
	90 to 150	107	35.1	40.5 ± 11.2	
	>150	97	31.8	38.8 ± 11.2	
	*TP53* status				<0.001
	Wild-type	92	30.2	33.7 ± 12.2	
	Mutant	213	69.8	40.4 ± 11.2	
	Oncogenic signature				<0.001
	C-class	193	63.3	40.7 ± 11.2	
	M-class	96	31.5	34.9 ± 12.3	
	NA	16	5.2	30.3 ± 8.6	

^#^For variables analyzed as numeric, the value shown is the regression coefficient for the change in MATH per change in the variable, ± the standard error of the coefficient.

^†^ *p*-Values for linear analyses with MATH as outcome variable, omitting cases where data was not available for that characteristic.

*p-Values for analysis as numeric variables; MATH values by groups also shown.

^‡^Oral also includes oral tongue, lip, alveolar ridge, buccal mucosa, floor of mouth, and hard palate; oropharynx also includes base of tongue and tonsil.

NA, not available.

Given the importance of HPV in HNSCC, we examined whether adjusting for HPV status, by including it as a second predictor variable in each of the linear models, would affect any of the apparent relations between MATH values and clinical characteristics (Table 3). After this adjustment for HPV, the relation between MATH value and anatomic tumor site was no longer significant; the predominance of HPV-positive tumors in the oropharynx (S1 Table) apparently accounted for the low MATH values seen for oropharyngeal tumors in Table 2. This adjustment for HPV exposed relations between MATH value and age and N classification, while other clinical characteristics associated with MATH value in univariate analyses (Table 2) maintained significance. MATH value was not significantly related to T classification or to TNM stage, even after adjustment for HPV status.

**Table 3:** Relations of clinical and molecular characteristics to MATH values, adjusting for HPV status.

Category	Characteristic	MATH Value ± SD (Number of Cases)		*p*-Value^#^
		HPV-Negative	HPV-Positive	
Clinical characteristics	Age^†^			0.046
	<56 y	41.4 ± 10.2 (68)	35.0 ± 15.4 (15)	
	56 to 65 y	40.2 ± 12.3 (80)	30.0 ± 9.5 (12)	
	>65 y	38.4 ± 10.8 (94)	37.2 ± 14.8 (9)	
	Prior cancer diagnosis			0.042
	No	39.8 ± 11.3 (232)	31.5 ± 11.1 (32)	
	Yes	40.6 ± 7.7 (10)	52.9 ± 17.1 (4)	
	Neoadjuvant history			0.026
	No	39.7 ± 11.1 (236)	32.8 ± 12.5 (34)	
	Yes	45.6 ± 12.9 (6)	52.0 ± 22.2 (2)	
	Tumor histologic grade			0.005
	G1	33.4 ± 11.3 (22)	24.9 (1)	
	G2, G3, or G4	40.3 ± 10.9 (215)	34.7 ± 13.8 (32)	
	LVI			0.002
	Absent	38.4 ± 10.8 (115)	32.4 ± 13.6 (11)	
	Present	44.2 ± 11.5 (57)	32.7 ± 5.1 (2)	
	N classification^†^			0.039
	0	38.0 ± 11.5 (107)	33.5 ± 15.6 (13)	
	1	39.7 ± 10.8 (34)	39.6 ± 17.3 (5)	
	2	41.5 ± 10.8 (95)	32.8 ± 11.3 (17)	
	3	44.5 ± 9.3 (5)	29.0 (1)	
Molecular characteristics	*TP53* status			
	Wild-type	36.3 ± 12.4 (42)	33.0 ± 12.6 (35)	0.009
	Mutant	40.6 ± 10.8 (200)	65.1 (1)	
	Oncogenic signature			0.001
	C-class	41.5 ± 10.3 (170)	38.8 ± 14.1 (11)	
	M-class	36.7 ± 12.4 (65)	32.0 ± 13.4 (21)	

Analysis restricted to the 278 tumors with data on HPV status. In bivariate linear analyses including HPV status, MATH values were not significantly related to gender (3.0 units higher in males, *p* = 0.055), the number of mutated loci (3.2 units higher per 10-fold increase in number of loci, *p* = 0.092), T classification (1.1 units higher per T class, *p* = 0.12), nodal extracapsular spread (3.6 units higher, *p* = 0.12, among pN+ nodes), or any of the following (*p* > 0.15): ethnicity, race, tumor site, alcohol use, tobacco use, TNM stage, tumor margin status, or perineural invasion.

^#^For relation of MATH to indicated characteristic in bivariate linear analysis with HPV status.

^†^Analyzed as numeric; age groups for display.

We also examined whether tumor MATH values might provide information about the likelihood of regional metastases to lymph nodes. Among 194 patients with HPV-negative tumors whose cervical lymph nodes were examined pathologically, those with low-heterogeneity tumors were significantly less likely to have disease that had spread to lymph nodes. Of the 64 patients with low-heterogeneity tumors, 42% (27) had positive nodes, versus 67% (87) of the 130 patients with high-heterogeneity (high MATH value) tumors (odds ratio, 2.76; 95% CI, 1.43 to 5.38; *p* = 0.001, Fisher exact test).

### High Intra-Tumor Heterogeneity Was Related to Increased Mortality

**Fig 4:**
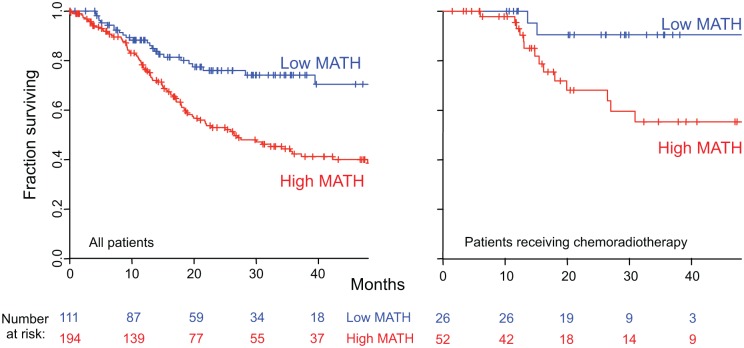
Relation of intra-tumor heterogeneity to overall survival in HNSCC. Kaplan-Meier curves for patients with high- or low-heterogeneity tumors, based on the MATH-value cutoff of 32 used in a previous study [31]. Left, for all 305 patients. Right, for 78 patients receiving chemoradiation as primary therapy or as an adjuvant to surgery. Corresponding HRs, confidence intervals, and *p*-values are in the main text.

**Fig 5:**
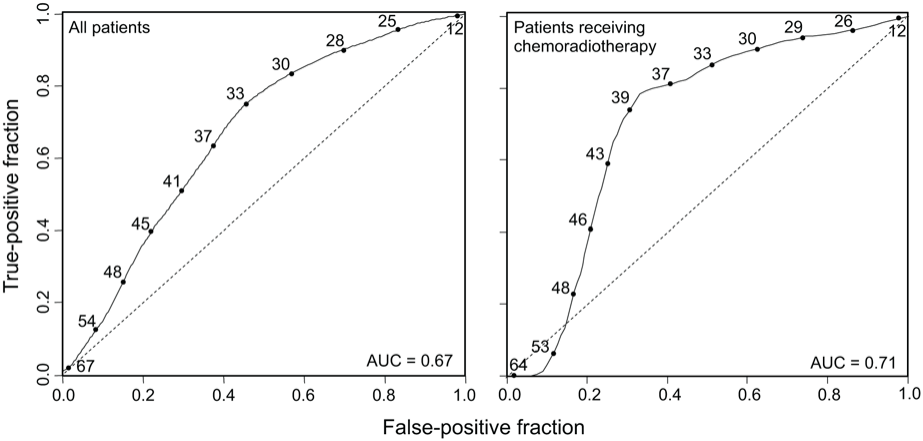
MATH receiver operating characteristic curves. Time-dependent ROC curves (solid lines) evaluated at 3-y survival, obtained by the nearest neighbor method of Heagerty et al. [44], with a smoothing span of 0.1. Curves show the relation between sensitivity (true-positive fraction) and specificity (1 − false-positive fraction) as the MATH value used to distinguish high- from low-heterogeneity tumors (values shown along the curves) is altered. Dashed lines are lines of identity. Left, ROC curve for all 305 patients (95% CI for AUC, 0.60 to 0.74). Right, ROC curve for 78 patients receiving chemoradiation as primary therapy or as an adjuvant to surgery (95% CI for AUC, 0.54 to 0.82).

MATH, taken as a continuous variable, was strongly related to overall survival; each 10% increase in MATH value corresponded to an 8.8% increased hazard of death (95% CI, 3.3% to 15% increased hazard per 10% increase in MATH; *p* = 0.001). For comparison with the initial study of MATH and survival in HNSCC [31], we used the previous MATH-value cutoff of 32 to distinguish high- from low-heterogeneity tumors; 194 tumors (63.6%) were thus classified as high heterogeneity. Patients with high- versus low-heterogeneity tumors had double the hazard of death (HR, 2.18; 95% CI, 1.44 to 3.30; *p* < 0.001; Fig. 4, left). The tradeoff between specificity and sensitivity for 3-y survival predictions as the high/low MATH-value cutoff varied is illustrated in the ROC curve of Fig. 5 (left). A relation of intra-tumor heterogeneity to outcome has long been suspected, particularly in patients treated with systemic therapy [1,3–5], and chemoradiation is frequently used to treat advanced primary HNSCC [46]. We thus examined the relation between MATH value and survival specifically in patients identified as receiving chemoradiation as primary therapy or as an adjuvant to surgery. The relation of intra-tumor heterogeneity to outcome was also seen in this subset of 78 patients (HR, 5.2; 95% CI, 1.2 to 23; *p* = 0.03; Fig. 4, right; ROC curve, Fig. 5, right), validating our prior results [30].

### The Relation of MATH to Mortality Was Not Due to Its Relation to HPV or to Other Clinical Characteristics

The relation of MATH values to clinical characteristics that are themselves associated with survival raised the question of whether the relation of MATH value to outcome simply represented its relation to those other characteristics. We thus examined the joint relations of MATH value and its associated characteristics to outcome, in bivariate and multivariate Cox proportional hazards analyses.

**Fig 6:**
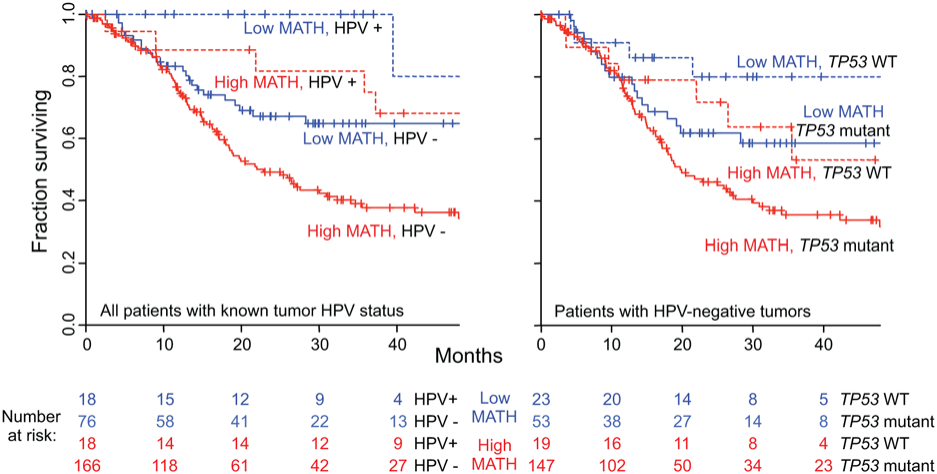
Combined relation of MATH and HPV or *TP53* mutation status to overall survival. Left, joint relation of intra-tumor heterogeneity and HPV status to overall survival in HNSCC among 278 patients whose tumor HPV status had been assessed by TCGA molecular criteria (see Methods). Kaplan-Meier curves are for the indicated combinations of HPV and MATH status; high MATH is MATH > 32. Blue, low MATH value; red, high MATH value; dashed lines, HPV-positive; solid lines, HPV-negative. Cox proportional hazards analysis: high/low MATH HR, 1.85 (95% CI, 1.22 to 2.80; *p* = 0.004, Wald test); HPV-positive/-negative HR, 0.36 (95% CI, 0.18 to 0.74; *p* = 0.006). Right, joint relation of intra-tumor heterogeneity and *TP53* mutation status to overall survival in HNSCC among 242 patients with HPV-negative tumors. Kaplan-Meier curves are for the indicated combinations of *TP53* and MATH status. Dashed lines, wild-type (WT) *TP53*; solid lines, mutant *TP53*. Cox proportional hazards analysis: high/low MATH HR, 1.62 (95% CI, 1.04 to 2.52; *p* = 0.031, Wald test); mutant/wild-type *TP53* HR, 1.75 (95% CI, 1.002 to 3.05; *p* = 0.049).

Despite the strong association of HPV-positive tumors with low MATH values (Table 2), both MATH value and HPV status were significantly related to overall survival in bivariate Cox proportional hazards analysis. Fig. 6 (left) shows survival curves for combinations of high/low MATH values and HPV status. This joint relation of MATH value and HPV status to outcome supports a role of intra-tumor heterogeneity in HNSCC mortality independent of HPV status [31].

We then examined whether other clinical characteristics that were associated both with MATH values and with overall survival might account for the relation of MATH to survival. After adjustment for HPV status, only the clinical characteristics of age, tumor grade, and N classification were significantly associated with both MATH values (Table 3) and overall survival (S2 Table). To evaluate whether these correlated clinical characteristics might account for the relation of MATH to outcome, we performed multivariate survival analysis incorporating these three clinical characteristics, HPV status, and MATH value. MATH value remained significantly related to overall survival in this analysis (S3 Table). Thus, MATH value is associated with survival after adjustment for correlated clinical characteristics.

### The Relation of MATH to Mortality Was Not Due to Its Relation to Other Molecular Characteristics of the Tumors

In univariate analyses, MATH values were related to three other molecular characteristics of the tumors: the number of somatic mutations in the exome (a measure of tumor mutation rate [33]), *TP53* mutation status [38], and oncogenic signature class [12] (Table 2, bottom). We thus examined whether the relations of these molecular characteristics to MATH might account for its relation to overall survival.

First, although a tumor’s mutation rate as measured by its number of exome mutations was associated with MATH values in univariate analysis (Table 2), this relation was no longer significant after adjustment for HPV status (Table 3), and mutation rate was not itself significantly related to overall survival (Table 1), particularly after adjustment for HPV (S2 Table). These results ruled out mutation rate as an explanation for the relation of MATH to outcome.

Second, MATH values were significantly higher in HPV-negative tumors that harbored *TP53* mutations than in HPV-positive/*TP53* wild-type tumors (Table 3), and as expected [38], mutated *TP53* was significantly related to diminished overall survival (HR, 2.61; 95% CI, 1.67 to 4.07; *p* < 0.001). As shown in Fig. 6 (right), however, both MATH and *TP53* mutation status were significantly related to survival among patients with HPV-negative tumors. (Only one HPV-positive tumor bore a mutation in *TP53*.) Thus, both high MATH value and mutated *TP53* were associated with survival after adjustment for their relation to each other.

Third, a novel molecular classification based on frequently occurring DNA disruptions among multiple types of tumors, called the oncogenic signature [12], was related both to survival and to MATH value. Oncogenic signatures are genomic classifications based on over 3,000 TCGA tumors from multiple anatomic sites, with the major classes called “M” and “C.” Disruptions in M-class tumors are dominated by small mutations (single nucleotide variants and small indels), versus predominant CNAs in C-class tumors. Oncogenic signature class had a significant univariate relation to outcome, with better overall survival in patients with M-class tumors (Table 1). Furthermore, MATH value in M-class tumors was significantly lower than in C-class tumors, even when HPV status was taken into account (Table 3), while HPV-positive tumors were predominantly in the M class (HPV-positive tumors: 11 C-class, 21 M-class; HPV-negative tumors: 170 C-class, 65 M-class; *p* < 0.001, Fisher exact test). We examined the joint relation of oncogenic signature class, HPV status, and MATH value to outcome in Cox proportional hazards analysis. In this trivariate analysis, oncogenic signature class was no longer associated significantly with outcome (M-class/C-class HR, 0.88; 95% CI, 0.57 to 1.34; *p* = 0.54), while MATH value (high/low HR, 1.75; 95% CI, 1.11 to 2.72; *p* = 0.015) and HPV status (HVP-positive/-negative HR, 0.31; 95% CI, 0.13 to 0.71; *p* = 0.006) both remained significantly related to outcome. Thus, the relation of high MATH value to increased mortality is not due to its associations with the tumor molecular characteristics of mutation rate, *TP53* mutation, and oncogenic signature.

### MATH Contributes Clinically Useful Prognostic Information

Having found that the relation of high MATH value to increased mortality was not simply due to its relations to patient clinical characteristics or to other molecular characteristics of the tumors, we examined whether MATH could further aid in prognostication.

**Fig 7:**
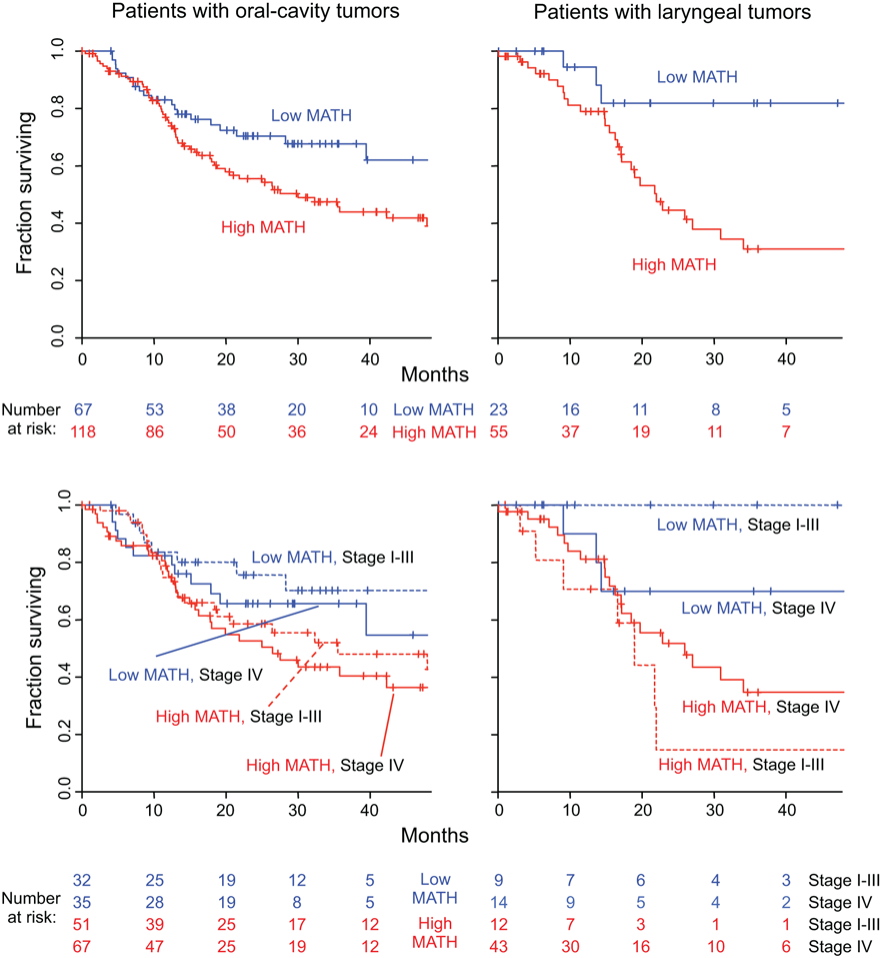
MATH value and mortality in patients with tumors in the oral cavity or the larynx. Left, patients with oral-cavity tumors; right, patients with laryngeal tumors. Top, Kaplan-Meier curves for all patients. Blue, low MATH; red, high MATH (MATH > 32). Cox proportional hazards analysis: oral cavity, high/low MATH HR, 1.69 (95% CI, 1.04 to 2.73; *p* = 0.033); larynx, high/low MATH HR, 3.55 (95% CI, 1.25 to 10.1; *p* = 0.018). Bottom, joint relation of MATH value and disease stage to survival; dashed, lines Stages I–III; solid lines, Stage IV. Cox proportional hazards analysis: oral cavity, high/low MATH HR, 1.67 (95% CI, 1.03 to 2.70; *p* = 0.037, Wald test); Stage IV/Stage I–III HR, 1.34 (95% CI, 0.87 to 2.08; *p* = 0.19); larynx, high/low MATH HR, 3.50 (95% CI, 1.18 to 10.4; *p* = 0.024); Stage IV/Stage I–III HR, 1.04 (95% CI, 0.44 to 2.49; *p* = 0.92).

We examined whether MATH could improve prognostication in oral-cavity or laryngeal tumors. HPV and its associated better prognosis is seldom involved at these anatomic sites [36], unlike oropharyngeal tumors, so that additional prognostic information beyond that provided by TNM staging [23] is needed. High versus low MATH value significantly distinguished outcomes in patients with tumors at either site (Fig. 7, top), even when TNM staging was taken into account (Fig. 7, bottom). These results support MATH as an additional prognostic variable for patients with tumors at those sites.

Another use of MATH could be in multivariate survival models that incorporate clinical and molecular characteristics to stratify patients by expected outcome for clinical trials or clinical decision-making. We thus examined MATH along with variables known to be associated with HNSCC outcome—HPV and *TP53* status, and seven standard clinical characteristics—in multivariate Cox proportional hazards analysis, which adjusts for the relations among all the predictors. In this multivariate analysis, MATH value, age, and smoking history were found to be significantly related to outcome (Table 4).

**Table 4:** Multivariate relation of MATH value and standard prognostic variables to overall survival.

Clinical or Molecular Characteristic	HR	95% CI		*p*-Value
		Low	High	
MATH > 32	1.76	1.13	2.74	0.013
Age (per year)	1.03	1.01	1.06	0.013
Non-smoker, or quit >15 y	0.52	0.30	0.89	0.018
N classification > 1	1.69	0.93	3.07	0.083
HPV-positive	0.39	0.10	1.59	0.19
Tumor grade > 1	1.49	0.67	3.29	0.33
T classification > 2	1.24	0.73	2.12	0.42
Stage IV	0.78	0.38	1.63	0.52
*TP53* mutant	1.17	0.62	2.20	0.64
Male gender	0.89	0.51	1.56	0.69

Results of multivariate Cox proportional hazards analysis for 261 patients (114 deceased) with complete information on the indicated variables. Confidence intervals and *p*-values are based on a bootstrap estimate (400 resamples) of the variance–covariance matrix. There was some evidence of non-proportional hazards for tumor grade (*p* = 0.082, chi-square test for trend of residuals with time); a model stratified by tumor grade gave similar results with no evidence of non-proportional hazards (*p* > 0.3 for all variables).

## Discussion

These results validate and substantially extend our previous finding [31] that high intra-tumor heterogeneity predicts decreased overall survival in patients with HNSCC. Even after accounting for clinical and molecular characteristics of patients and their tumors, the magnitude of the mortality hazard associated with high intra-tumor heterogeneity, as measured by MATH (Table 4), was comparable to that of hazards associated with established prognostic variables (Tables 1 and 4).

### What the Study Adds to Existing Research

**Intra-tumor heterogeneity and cancer mortality.** To our knowledge, this is the first large-scale demonstration based on data from multiple institutions that intra-tumor heterogeneity per se is clinically important in the prognosis of any type of cancer. Using identical criteria for including tumor-specific mutated loci, calculating MATH values, and distinguishing high- from low-heterogeneity tumors as in the previous single-institution study, the present study found a highly significant relation of high intra-tumor heterogeneity to outcome. In both studies, the univariate overall survival HR for high/low MATH was over 2 (previous study, 2.46; this study, 2.18), and a relation of high intra-tumor heterogeneity to decreased overall survival was seen among patients receiving chemoradiotherapy (HR in previous study, 4.1; this study, 5.2). We found this strong relation of intra-tumor heterogeneity to overall survival despite the limitations of these TCGA data. In particular, the data were not collected with this analysis of heterogeneity in mind, and the variety of institutions, head and neck tumor subsites, and treatment modalities might have been expected to minimize our ability to identify significant prognostic variables. Our results thus suggest that intra-tumor heterogeneity can have substantial clinical importance.

**Sources and consequences of intra-tumor heterogeneity.** The intra-tumor genetic heterogeneity captured by MATH may arise from either CNAs or from subclonal mutations. Although the present results do not distinguish CNAs from subclonality, they do shed some light on the consequences of high intra-tumor heterogeneity and the processes that promote it. High MATH values in tumors containing *TP53* mutations suggest that deficiencies in DNA-damage and apoptotic responses may create an environment that is favorable to the generation or maintenance of intra-tumor genetic heterogeneity. High MATH values in tumors with the molecular C-class oncogenic signature [12], even after adjustment for HPV status, support a stronger role of CNAs than of point mutations in developing or maintaining intra-tumor heterogeneity as measured by MATH. Nevertheless, heterogeneity per se, rather than CNAs, seems most closely related to HNSCC outcome, as the low univariate M-class/C-class HR became insignificant once MATH and HPV status were taken into account. The relation of high MATH value to LVI and nodal status suggests that intra-tumor heterogeneity may foster regional metastasis. The relation of high MATH value to decreased survival in patients receiving cytotoxic therapy supports selection of preexisting resistant cancer cells by therapy [1,13] or the presence in heterogeneous tumors of subpopulations that provide resistance or promote the growth of the rest of the tumor [48] as mechanisms for evading such therapy. As these initial findings are expanded by further study on the mechanisms underlying the development of intra-tumor heterogeneity, it might become possible to turn those mechanisms into therapeutic targets.

**Prognostic variables in HNSCC.** These results highlight important issues to address in HNSCC prognostic models. First, some clinical characteristics related to overall survival (Table 1) are often difficult or impossible to evaluate in clinical practice in patients with low-T/high-N disease, where a tumor biopsy is performed to help choose definitive therapy. If therapy does not include surgical tumor excision or neck dissection [46], information on tumor margins, perineural invasion, LVI, and nodal extracapsular spread will be incomplete or unavailable. A MATH value, obtained from WES of a few milligrams of a tumor, does not face this limitation as a biomarker. Second, the close relations among *TP53* mutation status, HPV status, MATH value, and clinical characteristics mean that care must be taken in interpreting and using prognostic models in HNSCC. For example, the lack of statistical significance of N classification, HPV status, and *TP53* mutation in the ten-variable multivariate analysis shown in Table 4 does not mean that they are irrelevant to outcome; each of these characteristics bears a significant univariate relation to outcome in HNSCC (Table 1), and their apparent lack of significance in the multivariate model might simply represent the difficulty in unraveling the individual contributions of multiple highly correlated variables, particularly with only 36 HPV-positive tumors.

### Strengths and Limitations of This Study

**Strengths.** This study was designed as a validation test of the relation between mortality and MATH that we had found in a previous smaller, single-institution study. We used the previous methods for calculating MATH values, and the previous MATH cutoff between high- and low-heterogeneity tumors, without any attempt to optimize for the present data. The large number of patients and the multiple institutions represented in the TCGA data on HNSCC provided a stringent test of our previous findings, and allowed us to adjust for many clinical and molecular variables in our analyses of overall survival. Thus, the association of high intra-tumor heterogeneity in HNSCC with increased mortality has been validated insofar as possible with this type of retrospective analysis.

**Limitations.** To define the usefulness of MATH in HNSCC and to extend similar analyses to other types of cancer, further work is needed to overcome several limitations of the present study. One issue is how MATH is measured in practice. In both this study and the previous report [31], MATH values were determined from WES data obtained with a consistent set of methods, from tumor processing through exome capture (and thus breadth of genomic sequencing coverage) to WES (at similar depth of sequencing) and calling of somatic mutations. As discussed previously [30], different combinations of technologies might lead to different or less reliable MATH values, in particular if a lower breadth of coverage limits the number of tumor-specific mutations found or if calling methods for somatic mutations or handling of loci with low MAFs differs from the methods in the present study. Also, given that the precision of determining a tumor’s MATH value depends on its number of tumor-specific mutated genomic loci [30], exome capture for a type of cancer with lower mutation rates than HNSCC might not provide enough tumor-specific mutations, so that a larger fraction of the genome might need to be sequenced.

Another issue is that the simplicity of the formula for calculating MATH values from WES data masks important aspects of underlying tumor biology. The MAFs observed in a bulk tumor DNA sample are determined by several factors: the “impurity” arising from normal DNA in non-cancer cells, the cancer-cell genomic ploidy arising from large-scale gain or loss of chromosomal segments or smaller-scale CNAs, and mutations specific to genetically distinct subclones within the tumor. Several methods have been developed to untangle these contributions [26–29]. The MATH measure of heterogeneity uses the median MAF value of the tumor as a first-order correction for “impurity,” and it combines heterogeneity arising from ploidy/CNAs and subclones, as both can contribute to the width of the distribution of a tumor’s MAF values [30]. More precise corrections for impurity and separate handling of ploidy and subclonal tumor inheritance patterns might ultimately lead to better measures of intra-tumor heterogeneity, but for now MATH provides a simple measure, closely related to mortality in HNSCC, that can be determined as soon as tumor-specific mutations have been called and MAF values are available.

Finally, several limitations of the TCGA clinical data need to be recognized. First, the TCGA requirement for tumor mass adequate for multiple analyses biases this dataset toward larger, surgically treated tumors and underrepresents the increasingly important HPV-positive HNSCC that often presents with low-T/high-N pathology [40] (see S1 Text); of 305 tumors, only 24 (three HPV-positive) were T1. Second, our use of statistical models to account for the contribution of HPV status to other clinical variables and to outcome might not properly capture the different biological bases and clinical history of HPV-positive and HPV-negative HNSCC. Coefficients in analyses that take HPV status into account in this way are necessarily weighted toward the more prevalent HPV-negative cases, and there were too few HPV-positive cases to allow analysis of the HPV-positive subset or of statistical interaction coefficients involving HPV status. Third, although much information about clinical treatments and outcomes was available from TCGA, these data were not collected prospectively, and many cases lacked complete treatment annotations. Thus, we could not, for example, resolve the important issue of whether high heterogeneity predicts shorter survival in patients treated solely with surgery. Finally, although Table 4 clearly demonstrates that MATH has a prognostic significance similar to that of accepted outcome markers in HNSCC, this particular multivariate model should not be used for clinical prognostication. Prospective study of homogeneously treated HNSCC at specific head and neck subsites, with appropriate model validation, will be required before such results can be used in clinical trials or in routine clinical practice. Further analysis of MATH and outcome specifically in HPV-positive oropharyngeal squamous cell carcinoma is particularly needed.

### Implications

The strong relation of higher intra-tumor heterogeneity as measured by MATH to decreased overall survival means that MATH should be considered a biomarker in HNSCC. The limitations of the present study, noted above, should be addressed by analyzing tumor specimens already collected in prospective clinical studies or by incorporating MATH analysis into future studies. Once validated in this way, MATH values will be able to provide a simple high/low heterogeneity characterization (Figs. 4, 6, and 7) or a continuous measure of intra-tumor heterogeneity (Fig. 5) in models designed for prognostication or for clinical trial designs that require identifying patients who are at either particularly high or low risk of succumbing to disease under current standards of care. In particular, with its relation to outcome following chemoradiation (Fig. 4, right) and its joint relation with HPV status to outcome (Fig. 6, left), MATH should be useful in clinical trials on de-intensification of organ-preservation therapy for oropharyngeal cancer, in which chemoradiation is a standard of care and HPV status is already considered in trial design [36]. In HPV-negative HNSCC, the relation of MATH to nodal involvement suggests that MATH might assist clinical studies in evaluating the need for cervical node dissection in patients with low-T/cN0 oral cancer, a decision presently based on tumor depth and sentinel node mapping [49]. Furthermore, MATH adds usefully to TNM staging in prognosticating overall survival of patients having either oral-cavity or laryngeal tumors (Fig. 7). Consequently, MATH could direct the need for adjuvant therapy or identify candidates for laryngeal preservation protocols. As HPV status has helped stratify patients with oropharyngeal tumors according to prognosis [36,46], MATH may help stratify patients with tumors at these head and neck sites where HPV-positive tumors are infrequent.

More generally, MATH will be straightforward to apply clinically in other types of cancer, for there is nothing specific to HNSCC in the underlying analysis of exome sequence data. Unlike approaches to measuring intra-tumor heterogeneity that require pre-identification of subclone markers [17,18], detailed analysis of SNP arrays [26,50], or analysis of multiple portions down to single cells of a tumor [13,20,22,25], MATH calculations require no information beyond a list of tumor-specific mutations and their MAFs, derived directly from a patient’s tumor and normal DNA. Thus, as WES enters the practice of clinical oncology [8,32], MATH will provide a novel and straightforward way to incorporate information about intra-tumor heterogeneity into clinical research and practice.

### Conclusions

Intra-tumor heterogeneity per se can be prognostically important in cancer. MATH, a novel measure of intra-tumor genetic heterogeneity, has a prognostic relation to outcome comparable to that of accepted biomarkers in HNSCC clinical oncology, adding information beyond that provided by other patient and tumor characteristics. The success in relating MATH to outcome in HNSCC supports its evaluation in other types of cancer.

## Supporting Information

S1 Data Clinical TCGA data used for this study. Data downloaded in tabular form from TCGA on October 8, 2013, then transferred to. xls spreadsheets. A key to TCGA institutions contributing patient data and tumor samples is also provided.
(XLS)

S1 Table Breakdown of clinical characteristics by HPV status. (XLS)

S2 Table Relations of clinical and molecular characteristics to outcome, adjusted for HPV status. (XLS)

S3 Table Multivariate survival analysis of MATH together with significantly associated clinical variables. (XLS)

S1 Text Analysis of data from the multiple contributing TCGA institutions and comparison against US nationwide data. (PDF)

S2 Text STROBE statement. (DOC)

## Abbreviations

AUC: area under the curve
CNA: copy-number aberration
HNSCC: head and neck squamous cell carcinoma
HPV: human papillomavirus
HR: hazard ratio
LVI: lymphovascular invasion
MAD: median absolute deviation
MAF: mutant-allele fraction
MATH: mutant-allele tumor heterogeneity
ROC: receiver operating characteristic
SD: standard deviation
TCGA: The Cancer Genome Atlas
TNM: tumor node metastasis
WES: whole-exome sequencing
