# Correction: Intra-tumor Genetic Heterogeneity and Mortality in Head and Neck Cancer: Analysis of Data from The Cancer Genome Atlas

**DOI:** 10.1371/journal.pmed.1001818

**Published:** 2015-03-31

**Authors:** The PLOS Medicine Staff

The second author’s name is spelled incorrectly. The correct name is: Aaron D. Tward. The correct citation is: Mroz EA, Tward AD, Hammon RJ, Ren Y, Rocco JW (2015) Intra-tumor Genetic Heterogeneity and Mortality in Head and Neck Cancer: Analysis of Data from The Cancer Genome Atlas. PLoS Med 12(2): e1001786. doi: 10.1371/journal.pmed.1001786
