# Correction: Intra-tumor Genetic Heterogeneity and Mortality in Head and Neck Cancer: Analysis of Data from The Cancer Genome Atlas

**DOI:** 10.1371/journal.pmed.1001844

**Published:** 2015-06-09

**Authors:** The PLOS Medicine Staff

## Notice of Republication

This article was republished on May 14, 2015, to correct an error in Table 4. The legend was missing from the TIFF version of the table which affected viewing the table on the PLOS Medicine site and all downloadable versions of the table itself. The PDF of the whole article was unaffected.
