# Supplementary material for: Intra-tumor Genetic Heterogeneity and Mortality in Head and Neck Cancer: Analysis of Data from The Cancer Genome Atlas
Source: PLoS Med. 2015 Feb 10;12(2):e1001786. doi: 10.1371/journal.pmed.1001786 (PMC4323109; doi:10.1371/journal.pmed.1001786)
Supplement: S1 Text — (PDF) [file pmed.1001786.s005.pdf]

Text S1: representativeness of TCGA HNSCC cases

The TCGA program was designed to subject hundreds of tumor samples from each of more than 20 cancer types to multiple molecular analyses:

http://cancergenome.nih.gov/cancersselected/biospeccriteria.

This requirement for adequate amounts of tissue would be expected to select against small tumors. Furthermore, samples were collected from a large number of participating institutions without a requirement that samples had been collected prospectively, collected specifically for TCGA, or with any attempt to provide a fair sample of clinical cases.

In principle, the nature of the TCGA program thus may lead to questions about how representative the cases are with respect to a standard clinical population, and whether differences among the contributing institutions might be skewing results. In practice, we found that the 305 TCGA cases from 14 institutions examined here represent a reasonable sample of HNSCC at anatomic sites typically treated with surgery, with a few cautions noted below.

Inter-institution differences

We were initially troubled by apparent differences in mortality among the 14 institutions contributing samples to this TCGA study, with a relative standard deviation of 75.5% in hazards among institutions if clinical and molecular characteristics were not taken into account (inter-institutional variance of Cox regression coefficients, 0.316; $p = 0.0004$, chi-square test on log-likelihood difference from null model; *coxme* package in R). There were also, however, significant differences in HPV prevalence and TP53 mutation status among institutions (not shown), presumably representing different demographics and HNSCC risk factors for patients seen at different institutions.

To investigate the apparent inter-institutional differences in mortality, we treated the 14 institutions as providing a random effect in Cox regression models, with other clinical and molecular variables treated as fixed effects. Simply adding tumor HPV status as a single fixed effect resulted in loss of statistical significance of the random effect of institutions ($p = 0.075$), indicating that differences in HPV prevalence alone accounted for two-thirds of the initial apparent variance among institutions (remaining inter-institutional variance of Cox coefficients, 0.10). Adding institutions as a random effect to the Cox survival model in Table 4 of the main text made no significant change to the fit of that 10-variable model ($p = 0.53$). When included with the variables of the Table 4 model, the random effect represented only a 21% standard deviation in hazards among institutions, with over 85% of the initial apparent variance among institutions accounted for by the clinical and molecular characteristics included in the Table 4 model (remaining inter-institutional variance of Cox coefficients, 0.037).

Stage and site distribution

We compared the distribution of disease stages and anatomic sites of the 305 TCGA cases examined in the present study against those for invasive carcinomas publicly available from the National Cancer Data Base (NCDB): https://oliver.facs.org/BMPub/Docs/, which includes

approximately 70% of cancer cases newly diagnosed in the USA:
https://www.facs.org/quality%20programs/cancer/ncdb.

We used NCDB data for the calendar year 2008, the median year of initial diagnosis of these TCGA cases, and combined the head and neck subsites reported in NCDB to match the subsite definitions of the present study. Although the NCDB data do not distinguish cases by histopathology, over 90% of head and neck cancers are squamous cell carcinoma [1], supporting use of the NCDB data as a reference.

All invasive head and neck tumors, numbers by site and stage

**NCDB, 2008**			Stage			
	I	II	III	IV	Total	% of total
Larynx	3120	1594	1806	2674	9194	30.0
Oropharynx	102	124	223	750	1199	3.9
Tonsil	252	387	1046	2966	4651	15.2
Total OP	354	511	1269	3716	5850	19.1
Lip	648	129	40	65	882	2.9
Gum/mouth	1016	634	368	1571	3589	11.7
FOM	511	246	148	690	1595	5.2
Tongue	1746	1015	1276	3753	7790	25.4
Total Oral	3921	2024	1832	6079	13856	45.2
Hypo	98	211	332	1085	1726	5.6
Total	7493	4340	5239	13554	30626	
% of total	24.5	14.2	17.1	44.3		

**TCGA**			Stage			
	I	II	III	IV	Total	% of total
Larynx	0	9	12	57	78	25.6
OP	3	5	6	25	39	12.8
Oral	14	39	30	102	185	60.7
Hypo	0	0	0	3	3	1.0
Total	17	53	48	187	305	
% of total	5.6	17.4	15.7	61.3		

As expected from the TCGA tissue-mass requirements, Stage I cases were under-represented in the TCGA data, with only 5.6% of TCGA cases Stage I, versus 24.5% in NCDB.

We restricted further analyses to cases having Stages II, III, or IV. Among those higher-stage cases, two anatomic sites treated less frequently by surgery (oropharynx and hypopharynx) were correspondingly under-represented in TCGA: oropharyngeal sites represented 12.5% of Stage II-IV TCGA cases versus 23.8% in NCDB; 1.0% of Stage II-IV TCGA cases were hypopharyngeal, versus 7.0% in NCDB.

The distributions among stages II,III and IV was indistinguishable between TCGA and NCDB for either oropharyngeal, hypopharyngeal, or oral-cavity cases (Fisher exact test; $p = 0.48$, 1, 0.73, respectively). For laryngeal cases, Stage IV cases were over-represented in TCGA relative to Stages II and III ($p < 0.0001$); this is presumably related to standard-of-care therapy of laryngeal cancer, where chemoradiation rather than surgical excision is used if there is hope of maintaining organ preservation [1]. If attempts at organ preservation were more likely with Stage II or III versus Stage IV laryngeal cancer, tumors from such cases would have been less available for TCGA.

Percent of cases by Stage within each site, limited to Stage II-IV cases

**NCDB, 2008**		Stage	
	II	III	IV
Larynx	26.2	29.7	44.0
Oropharynx	11.3	20.3	68.4
Tonsil	8.8	23.8	67.4
Total OP	9.3	23.1	67.6
Lip	55.1	17.1	27.8
Gum/mouth	24.6	14.3	61.1
FOM	22.7	13.7	63.7
Tongue	16.8	21.1	62.1
Total Oral	20.4	18.4	61.2
Hypo	13.0	20.4	66.6

**TCGA**		Stage	
	II	III	IV
Larynx	11.5	15.4	73.1
OP	13.9	16.7	69.4
Oral	22.8	17.5	59.6
Hypo	0.0	0.0	100.0

HPV status

TCGA has reported that the prevalence of HPV positivity at oropharyngeal and at other head and neck sites in TCGA cases is similar to known prevalences [2].

Summary

Having samples from 14 institutions provided the desired effect of averaging out inter-institutional differences in patient demographic, clinical and molecular characteristics. The TCGA description of their HNSCC cases as "representative of a surgical case series with predominantly oral cavity and laryngeal tumors" [2] is reasonable with respect to overall clinical experience in the USA, with under-representation of low-stage cases but generally balanced representation of Stage II, III and IV disease at sites treated surgically. Overall HPV prevalence within anatomic tumor sites was similar to expectations from the literature. Results within individual head and neck anatomic sites are thus expected to be reasonably representative of higher-stage HNSCC cases treated surgically in the USA.

1. Marur S, Forastiere AA (2008) Head and neck cancer: changing epidemiology, diagnosis, and treatment. Mayo Clin Proc 83: 489-501.
2. The Cancer Genome Atlas Research Network (2014) Comprehensive genomic characterization of head and neck squamous cell carcinomas. Nature in the press.
