# Supplementary material for: Intra-tumor Genetic Heterogeneity and Mortality in Head and Neck Cancer: Analysis of Data from The Cancer Genome Atlas
Source: PLoS Med. 2015 Feb 10;12(2):e1001786. doi: 10.1371/journal.pmed.1001786 (PMC4323109; doi:10.1371/journal.pmed.1001786)
Supplement: S2 Text — (DOC) [file pmed.1001786.s006.doc]

STROBE Statement—checklist of items that should be included in reports of observational studies

|  | Item No | Recommendation |
| --- | --- | --- |
| **Title and abstract** | 1 | (*a*) Indicate the study’s design with a commonly used term in the title or the abstract  **Abstract, Methods and Findings: Clinical and WES data were obtained from The Cancer Genome Atlas in October 2013 for 305 patients with head and neck squamous cell carcinoma (HNSCC), from 14 institutions … we found in this retrospective analysis** |
|  |  | (*b*) Provide in the abstract an informative and balanced summary of what was done and what was found **Abstract, Methods and Findings: we found in this retrospective analysis a substantial relation of high MATH to decreased overall survival (Cox proportional hazards analysis: hazard ratio for high/low heterogeneity, 2.2; 95% CI 1.4 to 3.3). This relation of intra-tumor heterogeneity to survival was not due to its associations with other clinical or molecular characteristics, including age, human papillomavirus status, tumor grade and *TP53* mutation, and N classification. MATH improved prognostication over that provided by traditional clinical and molecular characteristics, maintained a significant relation to survival in multivariate analyses, and distinguished outcomes among patients having oral-cavity or laryngeal cancers even when standard disease staging was taken into account.** |
| Introduction | | |
| Background/rationale | 2 | Explain the scientific background and rationale for the investigation being reported  **Introduction: High intra-tumor heterogeneity has long been hypothesized to lead to worse clinical outcome… (through) … we were limited in our ability to establish separate relations of MATH and HPV status to outcome due to the small sample size.** |
| Objectives | 3 | State specific objectives, including any prespecified hypotheses  **Introduction, at end: To examine whether this relation between intra-tumor heterogeneity and mortality could be generalized, we analyzed data on HNSCC from The Cancer Genome Atlas (TCGA) [37]. These open-access clinical and WES data provided an independent, large, multi-institutional validation data set for testing the relation of MATH to outcome. We examined the relations of MATH values to standard clinical variables and to three molecular characteristics of HNSCC: HPV status, TP53 mutations [38], and oncogenic signature [12]. Using the same methods as in our previous work, we tested the hypothesis that intra-tumor heterogeneity, as measured by MATH, was related to mortality in HNSCC after accounting for these potentially associated clinical and molecular characteristics.** |
| Methods | | |
| Study design | 4 | Present key elements of study design early in the paper  **See answer to “objectives” item 3 above, expanded in Methods.** |
| Setting | 5 | Describe the setting, locations, and relevant dates, including periods of recruitment, exposure, follow-up, and data collection  **Methods: The de-identified, publicly available clinical data used in this study were those released by TCGA through October 8, 2013 … Initial pathologic diagnoses were made between 1992 and 2011 (median, 2008) … Analysis of the data from the multiple contributing TCGA institutions and comparison against nationwide data support this characterization of the data set (S1_Text). For clinical data analysis, follow-up times and vital status reported in the main patient data table were updated from the follow-up tables.** |
| Participants | 6 | (*a*) *Cohort study*—Give the eligibility criteria, and the sources and methods of selection of participants. Describe methods of follow-up  *Case-control study*—Give the eligibility criteria, and the sources and methods of case ascertainment and control selection. Give the rationale for the choice of cases and controls  *Cross-sectional study*—Give the eligibility criteria, and the sources and methods of selection of participants  **Methods: The de-identified, publicly available clinical data used in this study were those released by TCGA through October 8, 2013** |
|  |  | (*b*) *Cohort study*—For matched studies, give matching criteria and number of exposed and unexposed  *Case-control study*—For matched studies, give matching criteria and the number of controls per case |
| Variables | 7 | Clearly define all outcomes, exposures, predictors, potential confounders, and effect modifiers. Give diagnostic criteria, if applicable  **Methods, near end: overall survival (time between initial pathologic diagnosis and death). Methods, starting in second paragraph: TNM classifications were based on pathologic determinations where available. Disease staging was as reported by TCGA. Radiation or chemotherapy was identified as primary or adjuvant based on the “radiation_therapy,” “postoperative_rx_tx”, “targeted_molecular_therapy,” “regimen_indication” and “regimen_indication_notes” fields in the TCGA patient, drug, radiation and follow-up data tables. … Tumor-specific mutation data from WES were downloaded from the Broad Institute of MIT and Harvard [42], where WES had been performed [41].** |
| Data sources/ measurement | 8* | For each variable of interest, give sources of data and details of methods of assessment (measurement). Describe comparability of assessment methods if there is more than one group  **Methods, following the above: The steps in determining the MATH value of an individual tumor from the WES data were: 1) identifying genomic loci having tumor-specific somatic mutations, based on tumor-normal DNA comparisons; 2) tabulating the mutant-allele fractions (MAF, the fraction of DNA that shows the mutated allele at a locus) for mutated loci in that tumor; 3) determining the center and the width of the distribution of mutant-allele fractions among those loci; 4) taking the ratio of the width to the center of the distribution, expressed as a percentage**. |
| Bias | 9 | Describe any efforts to address potential sources of bias  **See analysis reported in S1_Text.** |
| Study size | 10 | Explain how the study size was arrived at  **Methods: The de-identified, publicly available clinical data used in this study were those released by TCGA through October 8, 2013 [39]. The data tables downloaded on that date, for 360 patients, are provided as S1 Data… Mutation data were available for 306 of the 360 patients having clinical data… All mutations in 1 tumor had MAF values below that cutoff, so that 305 cases remained for this study.** |
| Quantitative variables | 11 | Explain how quantitative variables were handled in the analyses. If applicable, describe which groupings were chosen and why  **Results, section “High Intra-Tumor Heterogeneity Was Related to Increased Mortality”: For comparison with the initial study of MATH and survival in HNSCC [31], we used the previous MATH-value cutoff of 32 to distinguish high- from low-heterogeneity tumors** |
| Statistical methods | 12 | (*a*) Describe all statistical methods, including those used to control for confounding  **Methods, starting from third-to-last paragraph: Relations of MATH to other clinical and molecular characteristics were examined by linear models. Relations of MATH and these characteristics to overall survival (time between initial pathologic diagnosis and death) were assessed by Cox proportional hazards analysis.**  **Receiver operating characteristic (ROC) curves for survival data were obtained by the nearest neighbor method of Heagerty et al. [43].** |
|  |  | (*b*) Describe any methods used to examine subgroups and interactions  **N/A, no special methods beyond definition of subgroups** |
|  |  | (*c*) Explain how missing data were addressed  **Tables 1,2, legends, “omitting NA cases.” Table 4, legend, 261 patients (114 deceased) having complete information on the indicated variables.** |
|  |  | (*d*) *Cohort study*—If applicable, explain how loss to follow-up was addressed  **N/A**  *Case-control study*—If applicable, explain how matching of cases and controls was addressed  *Cross-sectional study*—If applicable, describe analytical methods taking account of sampling strategy |
|  |  | (*e*) Describe any sensitivity analyses  **N/A** |

Continued on next page

| Results | | |
| --- | --- | --- |
| Participants | 13* | (a) Report numbers of individuals at each stage of study—eg numbers potentially eligible, examined for eligibility, confirmed eligible, included in the study, completing follow-up, and analysed. **See Study size, item 10 above** |
|  |  | (b) Give reasons for non-participation at each stage **See Study size, item 10 above** |
|  |  | (c) Consider use of a flow diagram **Simple enough not to require diagram** |
| Descriptive data | 14* | (a) Give characteristics of study participants (eg demographic, clinical, social) and information on exposures and potential confounders **Table 1 and S1_Table** |
|  |  | (b) Indicate number of participants with missing data for each variable of interest **See Tables 1, 2, S1_Table** |
|  |  | (c) *Cohort study*—Summarise follow-up time (eg, average and total amount) **Results, first paragraph: The median follow-up time for 174 patients still living at last record was 22.5 months (overall range, 0 to 142 months; inter-quartile range, 24.5 months), and the median time to death for the other 131 patients was 14.3 months (overall range, 0 to 211 months; inter-quartile range, 16.4 months).** |
| Outcome data | 15* | *Cohort study*—Report numbers of outcome events or summary measures over time. **Survival curves in Figs. 4,6,7** |
|  |  | *Case-control study—*Report numbers in each exposure category, or summary measures of exposure |
|  |  | *Cross-sectional study—*Report numbers of outcome events or summary measures |
| Main results | 16 | (*a*) Give unadjusted estimates and, if applicable, confounder-adjusted estimates and their precision (eg, 95% confidence interval). Make clear which confounders were adjusted for and why they were included Unadjusted: Results, section “High Intra-Tumor Heterogeneity Was Related to Increased Mortality: Patients with high- versus low-heterogeneity tumors had double the hazard of death (hazard ratio, HR, 2.18; 95% CI, 1.44 to 3.30; *p* = 0.0002; Fig.4, top). Adjusted, see Results, last paragraph: We thus examined MATH along with variables known to be associated with HNSCC outcome—HPV and *TP53* status, and 7 standard clinical characteristics—in multivariate Cox proportional hazards analysis, which adjusts for the relations among all these predictors. In this multivariate analysis, MATH value, age, and smoking history were found to be significantly related to outcome (Table 4). Also, Results section “The Relation of MATH to Mortality Was Not Due to Its Relation to Other Molecular Characteristics of the Tumors” |
|  |  | (*b*) Report category boundaries when continuous variables were categorized **See “quantitative variables” item 11 above; also legend to Fig.3** |
|  |  | (*c*) If relevant, consider translating estimates of relative risk into absolute risk for a meaningful time period **Survival fractions are displayed in Figs 4,6,7.** |
| Other analyses | 17 | Report other analyses done—eg analyses of subgroups and interactions, and sensitivity analyses **No interactions; subgroups based on clinical or molecular variables are examined throughout.** |
| Discussion | | |
| Key results | 18 | Summarise key results with reference to study objectives **Discussion, beginning: These results validate and substantially extend our previous finding [31] that high intra-tumor heterogeneity predicts decreased overall survival in patients with HNSCC. Even after accounting for clinical and molecular characteristics of patients and their tumors, the magnitude of the mortality hazard associated with high intra-tumor heterogeneity, as measured by MATH (Table 4), was comparable to that of hazards associated with established prognostic variables (Tables 1 and 4).** |
| Limitations | 19 | Discuss limitations of the study, taking into account sources of potential bias or imprecision. Discuss both direction and magnitude of any potential bias **Discussion, section “Strengths and Limitations of This Study,” subheading “Limitations”: To define the usefulness of MATH in HNSCC and to extend similar analyses to other types of cancer, further work is needed to overcome several limitations of the present study…** |
| Interpretation | 20 | Give a cautious overall interpretation of results considering objectives, limitations, multiplicity of analyses, results from similar studies, and other relevant evidence **Conclusion:** **Intra-tumor heterogeneity per se can be prognostically important in cancer. MATH, a novel measure of intra-tumor genetic heterogeneity, has a prognostic relation to outcome comparable to that of accepted biomarkers in HNSCC clinical oncology, adding information beyond that provided by other patient and tumor characteristics.** |
| Generalisability | 21 | Discuss the generalisability (external validity) of the study results **See under “Limitations” item 19 above.** |
| Other information | | |
| Funding | 22 | Give the source of funding and the role of the funders for the present study and, if applicable, for the original study on which the present article is based **Statement on Funding:**  **The National Institute of Dental and Craniofacial Research (R01 DE022087) and the Bacardi MEEI Biobank Fund. The funders had no role in study design, data collection and analysis, decision to publish, or preparation of the manuscript.** |

*Give information separately for cases and controls in case-control studies and, if applicable, for exposed and unexposed groups in cohort and cross-sectional studies.

**Note:** An Explanation and Elaboration article discusses each checklist item and gives methodological background and published examples of transparent reporting. The STROBE checklist is best used in conjunction with this article (freely available on the Web sites of PLoS Medicine at http://www.plosmedicine.org/, Annals of Internal Medicine at http://www.annals.org/, and Epidemiology at http://www.epidem.com/). Information on the STROBE Initiative is available at www.strobe-statement.org.
